# Supplementary material for: Hospital Mortality and Resource Implications of Hospitalisation with COVID-19 in London, UK: A Prospective Cohort Study
Source: Crit Care Res Pract. 2021 Jan 27;2021:8832660. doi: 10.1155/2021/8832660 (PMC7864763; doi:10.1155/2021/8832660)
Supplement: Supplementary Materials — Supplementary File 1. File name: Supplementary_file_1.pdf. Title: institutional setting. Description: details regarding the institutional setting of King's College Hospital and pandemic surge in the catchment area, including its temporal evolution with the use of heat maps. Supplementary File 2. File name: Supplementary_file_2.pdf. Title: definition of variables. Description: details regarding the definitions of variables used in the study. Supplementary File 3. File name: Supplementary_file_3.pdf. Title: statistical methods. Description: details regarding the statistical approach including the selection of variables and models. Description of the parametric regression equation. Additional figure describes the effect of each variable on the primary outcome of hospital mortality. Supplementary File 4. File name: Supplementary_file_4.pdf. Title: institutional surge planning and resource implications. Description: details regarding the reconfiguration of services implemented during the pandemic surge and the resources required. [file 8832660.f1.pdf]

Hospital mortality and resource implications of hospitalisation with COVID-19 in London, UK: a prospective cohort study

Supplementary File 1

Institutional Setting

King's College Hospital is a multi-site tertiary academic medical center in South London which serves a local population of approximately 700,000 inhabitants and is a regional referral center for trauma, cardiovascular medicine, neurosciences, hepatology and transplantation, hematology, and fetal medicine. It includes the main site at Denmark Hill (DH) and the Princess Royal University hospital (PRUH), a smaller district general hospital, as well as several outpatient facilities. Due to differences in ICU health record interface between the main DH site and the PRUH, only patients admitted to the main DH site were included in this study.

During the study period KCH admitted a large number of patients with suspected and confirmed COVID-19. The majority of these patients lived in the catchment area of the two hospital sites (DH and PRUH) and some were transferred from other institutions. The geographic and temporal evolution of the pandemic surge within KCH's catchment area in the month of March is shown in Figure S1.

Figure S1: Geographic and temporal evolution of the pandemic surge in the catchment area of KCH over four different weeks (Panels A-D). The Denmark Hill site is at the top left corner and the PRUH site at the bottom right.

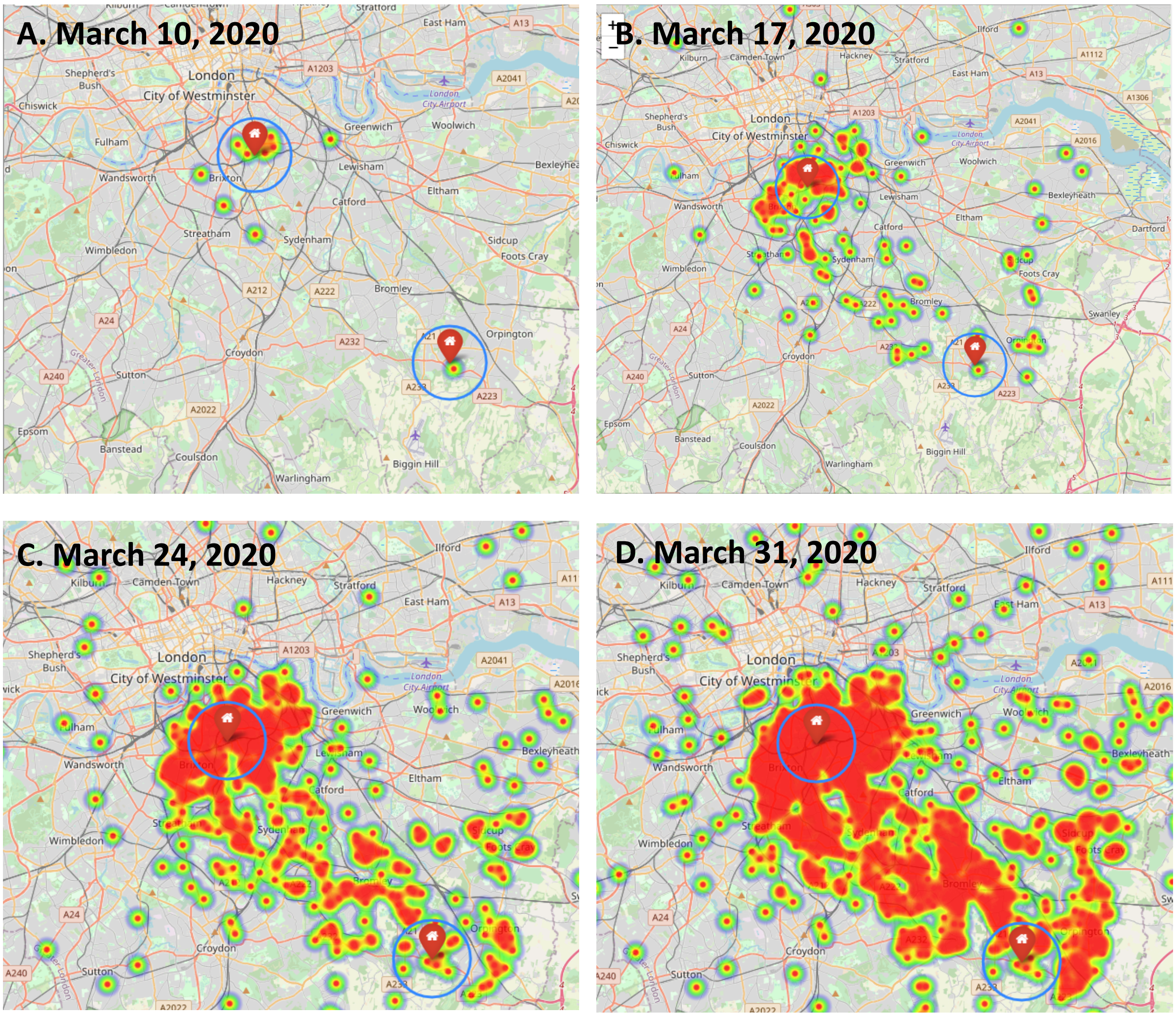

Page intentionally left blank

Hospital mortality and resource implications of hospitalisation with COVID-19 in London, UK: a prospective cohort study

Supplementary File 2

Definitions of variables

a. Laboratory results

All laboratory results were extracted manually from the Electronic Health Record (Sunrise EPR, Allscripts). We only used finalized and validated results. Reference values refer to those used in our laboratory. Cultures of biological samples (sputum, blood, urine) were considered positive when a specific microorganism was identified in the laboratory results. Results labelled as potential contamination were not included.

b. Imaging results

All imaging tests results were also extracted manually from the Electronic Health Record. We only used finalized and validated reports. All imaging tests in our institution are validated by specialist radiologists. Classification of findings adhered to the language used in the report.

c. Treatment limitations

Treatment limitation included the institution of any Treatment Escalation Plan, Do-Not-Resuscitate (DNR) orders or other limitation in the escalation of treatment. We did not differentiate between treatment limitations that were instituted during the hospital admission from any pre-existing decision such as Advanced Directives or Community DNR orders.

d. NEWS2 score

The National Early Warning Score (NEWS) 2 is an aggregate scoring system of physiological parameters that are routinely measured in hospitalized patients. The score is composed of the following:

i. respiratory rate
ii. peripheral oxygen saturation
iii. systolic blood pressure
iv. heart rate
v. level of consciousness or new confusion
vi. peripheral temperature

A score is assigned to each measurement, based on the degree of physiologic derangement and then aggregated and uplifted by 2 points for those who require supplemental oxygen.
An aggregate score of >4 warrants urgent medical review while a score >6 triggers urgent review by the critical care team. The NEWS2 score has been extensively validated [1,2].

e. SOFA score

The sequential organ failure assessment (SOFA) score was designed to describe quantitatively and objectively the degree of organ dysfunction and failure over time. Organ assessment occurs over six domains: Respiration, Coagulation, Liver function, Cardiovascular function, Central Nervous system function and Renal Function. Each domain is scored from 1 to 4, based on predetermined thresholds. The aggregate provides the SOFA score. The SOFA score has been extensively validated [3].

f. Index of Multiple Deprivation

Index of Multiple Deprivation (IMD) scores were assigned by linking residential address postcodes [4] registered on hospital admission to the corresponding neighborhood, also called a lower-layer super output area (LSOA) [5]. The IMD is the official measure of relative deprivation across LSOAs in England and it is calculated by combining and weighting 39 different indicators across seven distinct domains for every LSOA

[6]. As a neighborhood-level marker of socioeconomic status, it is correlated with behavioral risk factors, cardiovascular disease, malnutrition and overall health [7-11].

The IMD is based on 39 separate indicators, organized across seven distinct domains of deprivation which are combined and weighted as follows: Income (22.5%), Employment (22.5%), Health and disability (13.5%), Education, skills and training (13.5%), Crime (9.3%), Barriers to housing and services (9.3%), and Living environment (9.3%) [6].

The IMD is an overall measure of multiple deprivation experienced by people living in an area and is calculated for every LSOA, or neighborhood, in England. All neighborhoods are then ranked according to their IMD score relative to that of other areas. High ranking neighborhoods are given a rank of 1 and referred to as the 'most deprived' while the least deprived LSOA is ranked 32,844. There is no definitive threshold above which an area is described as 'deprived' and the IMD measures deprivation on a relative rather than an absolute scale.

Grouping of IMD values in quintiles was based on national distributions with use of predefined national cut-offs [12].

g. Hypoxemic respiratory failure

Hypoxemic respiratory failure was defined as an arterial partial pressure of oxygen (PaO2) less than 60 mmHg (8 kPa) or an arterial oxygen saturation below 90%, on room air [13].

h. Acute Kidney Injury (AKI)

Acute Kidney injury was categorized according to the KDIGO criteria. Stage 3 AKI was defined as a threefold increase in baseline creatinine value or as a serum creatinine ≥ 353.6 mmol/l or as initiation of renal replacement therapy [14].

i. Renal replacement therapy (RRT)

Renal replacement therapy included continuous modalities (veno-venous hemodiafiltration and similar), intermittent modalities (intermittent hemodialysis) and peritoneal dialysis. Patients received different types of RRT at different stages of their hospital stay, based on expert nephrological input.

References

1. Smith GB, Redfern OC, Pimentel MA, et al. The National Early Warning Score 2 (NEWS2). *Clinical medicine (London, England)* 2019; **19:** 260.
2. Royal College of Physicians. National Early Warning Score (NEWS) 2: Standardising the assessment of acute-illness severity in the NHS. https://www.rcplondon.ac.uk/projects/outputs/national-early-warning-score-news-2 (accessed May 2, 2020/.
3. Vincent JL, Moreno R, Takala J, et al. The SOFA (Sepsis-related Organ Failure Assessment) score to describe organ dysfunction/failure. On behalf of the Working Group on Sepsis-Related Problems of the European Society of Intensive Care Medicine. *Intensive Care Med* 1996; **22:** 707-10.
4. Open Geography P. Postcode to Output Area to Lower Layer Super Output Area to Middle Layer Super Output Area to Local Authority District (August 2019), 2019.
5. Data UMO. English Indices of Deprivation 2019 - LSOA Level, 2019.
6. Noble S, McLennan D, Noble M, et al. The English Indices of Deprivation 2019. https://assets.publishing.service.gov.uk/government/uploads/system/uploads/attachment_data/file/833947/IoD2019_Research_Report.pdf (accessed May 3, 2020/.
7. El-Sayed AM, Scarborough P, Galea S. Socioeconomic inequalities in childhood obesity in the United Kingdom: a systematic review of the literature. *Obes Facts* 2012; **5:** 671-92.
8. Hayden KE, Sandle LN, Berry JL. Ethnicity and social deprivation contribute to vitamin D deficiency in an urban UK population. *J Steroid Biochem Mol Biol* 2015; **148:** 253-5.
9. Kontopantelis E, Mamas MA, van Marwijk H, et al. Geographical epidemiology of health and overall deprivation in England, its changes and persistence from 2004 to 2015: a longitudinal spatial population study. *J Epidemiol Community Health* 2018; **72:** 140-7.
10. Lakshman R, McConville A, How S, Flowers J, Wareham N, Cosford P. Association between area-level socioeconomic deprivation and a cluster of behavioural risk factors: cross-sectional, population-based study. *Journal of Public Health* 2011; **33:** 234-45.
11. Ramsay SE, Morris RW, Whincup PH, et al. The influence of neighbourhood-level socioeconomic deprivation on cardiovascular disease mortality in older age: longitudinal multilevel analyses from a cohort of older British men. *J Epidemiol Community Health* 2015; **69:** 1224-31.
12. Ministry of Housing Communities & Local Government. English indices of deprivation 2019. https://www.gov.uk/government/statistics/english-indices-of-deprivation-2019 (accessed May 15 2020/.
13. Collins DW, Shehabi Y. Arterial Hypoxemia. In: Vincent J-L , Hall JB, eds. *Encyclopedia of Intensive Care Medicine.* Berlin, Heidelberg: Springer Berlin Heidelberg, 2012: 257-66.
14. Section 2: AKI Definition. *Kidney Int Suppl (2011)* 2012; **2:** 19-36.

Page intentionally left blank

Hospital mortality and resource implications of hospitalisation with COVID-19 in London, UK: a prospective cohort study

Supplementary File 3

Statistical methods

A. General approach

In our survival analysis, risk onset was considered to be February 25th, 2020, the date on which SARS-CoV-2 testing became available in our institution. Study entry for each patient was considered to be on the date of hospital admission for newly admitted patients and on the date of risk onset for patients who were already admitted. Follow-up continued until hospital discharge, death or the end of our study period on April 30th, 2020. Patients who were still alive and admitted to hospital after this date were right-censored.

We used the date of hospital admission instead of the date of symptom onset since it is not clear whether the trajectory and outcomes from COVID-19 are a function of time since onset of symptoms (which is subject to recall bias) or a function of time since reaching a certain degree of physiologic derangement. In the latter case, hospital admission represents a more objectively defined milestone in the pathophysiologic process of COVID-19.

We examined univariate association with survival probability using Kaplan-Meier estimators, as shown in Figures S2 and S3. We also fitted Cox proportional hazard models for each variable, without any covariates. In these unadjusted analyses, survival to hospital discharge or 30 days was associated with age, sex, ACCI, frailty, CRP and creatinine level, presence of chronic kidney disease (CKD) and diabetes, and dyspnea or fever as presenting complaints. It was not associated with ethnicity or level of deprivation. Further model development included a stepwise approach during which the empty Cox model (Model 0) was compared to one that included one additional variable, using likelihood ratio tests (LRT). Subsequent model refinement involved sequential inclusion of all variables and retainment based on the result of LRTs.

Figure S2: Kaplan-Meier curves for categories of age, sex, chronic kidney disease (CKD) and level of CRP

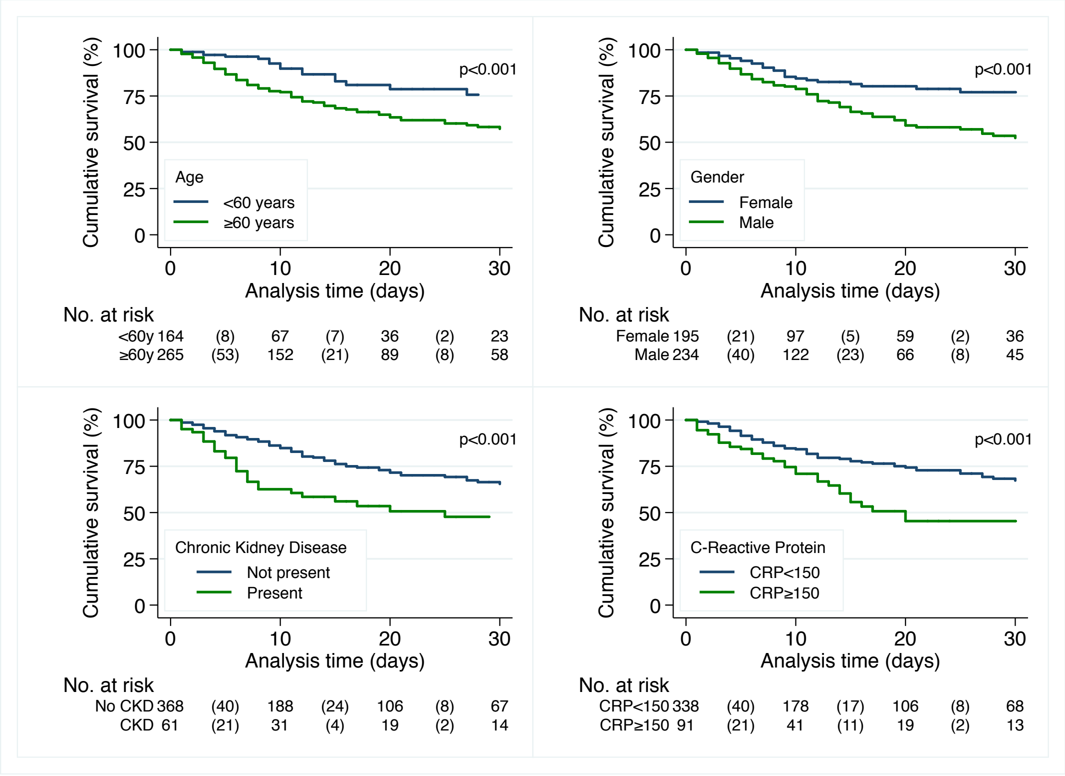

Figure S3: Kaplan-Meier curves for categories of deprivation and ethnicity

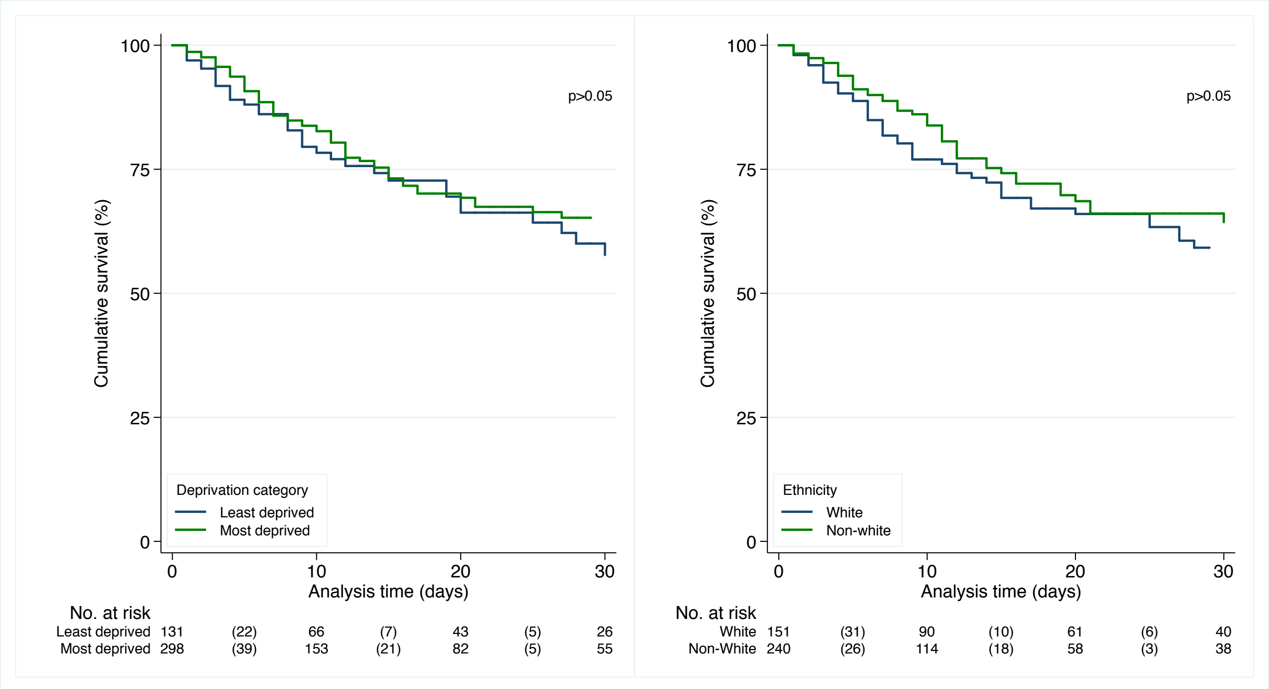

Age (in decades above 40 years), sex, baseline C-reactive protein level (CRP; in increments of 10mg above peak normal value of 5mg/L), presence of chronic kidney disease (CKD) and dyspnea as a presenting symptom were included in the preliminary model (Model 1). The preliminary model was then used to test interactions of all variables with age (Model 2), sex (Model 3) and time period in continuous (Model 4) and natural logarithm form (Model 5). As shown in Table S1, more complex models were not superior to Model 1 and hence the latter was used in subsequent analyses.

Table S1: Summary of different interaction model characteristics

	**Model 0**	**Model 1**	**Model 2**	**Model 3**	**Model 4**	**Model 5**
Number included	427	427	427	427	427	427
Log likelihood	-591.007	-549.847	-548.500	-549.154	-547.495	-547.794
LRT p-value (vs. Model 1)	<0.001	-	0.610	0.847	0.453	0.534
AIC	-	1109.693	1115.001	1116.308	1114.989	1115.588
BIC	-	1129.977	1151.512	1152.819	1155.557	1156.156

Graphical assessment of the PH assumption included plots of scaled Schoenfeld residuals against analysis time for continuous variables while for categorical variables it included plots of the log negative log Kaplan-Meier estimator $-ln\left[-ln\left\{\hat{S}(t)\right\}\right]$ against the natural logarithm of analysis time $ln(t)$, as shown in Figures S4 and S5.

Figure S4: Test of proportional hazards assumption for the continuous variables age and CRP

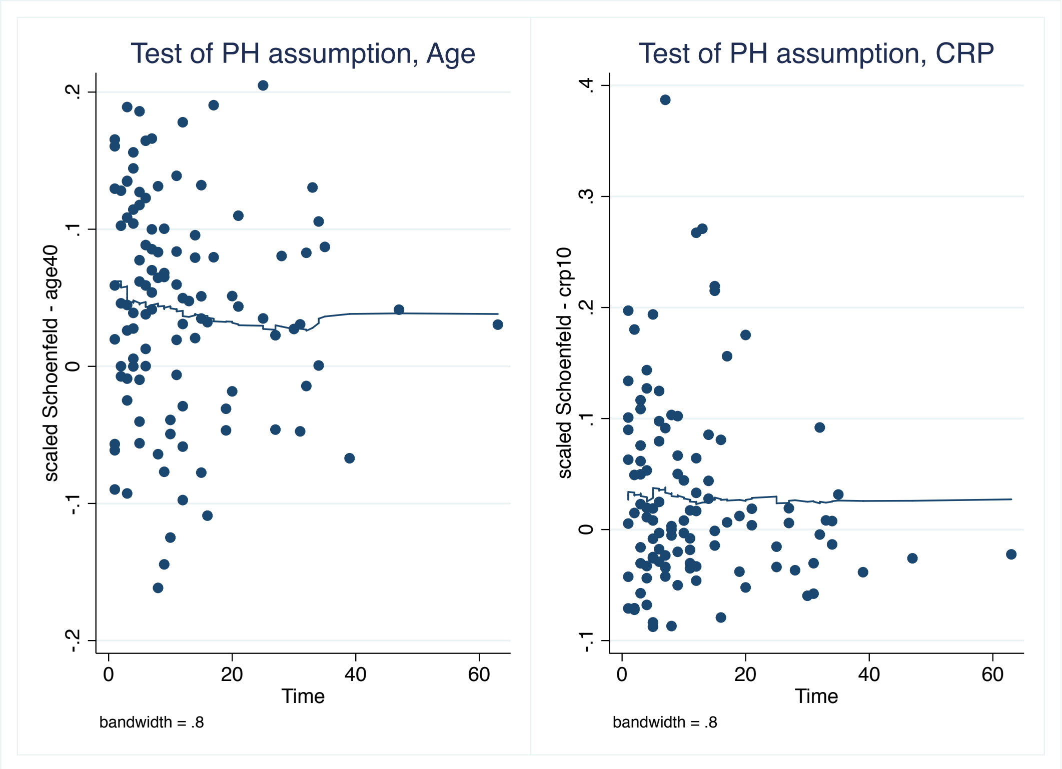

Figure S5: Test of proportional hazards assumption for categorical variables sex, chronic kidney disease (CKD), and dyspnea

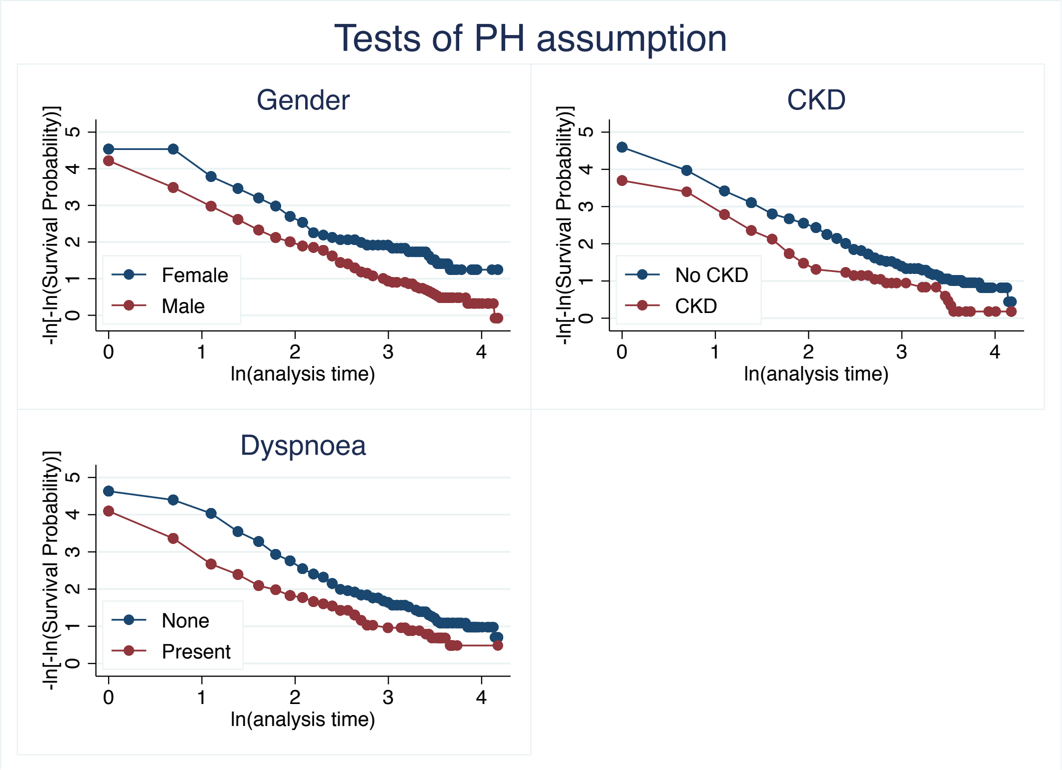

The functional form of included covariates was assessed using plots of Martingale residuals and, as a result, age was included as in quadratic form in the final model (Figure S6).

Figure S6: Martingale residuals for two forms of the variable "age": regular and squared. The squared form was preferred due to linearity and included in the final model.

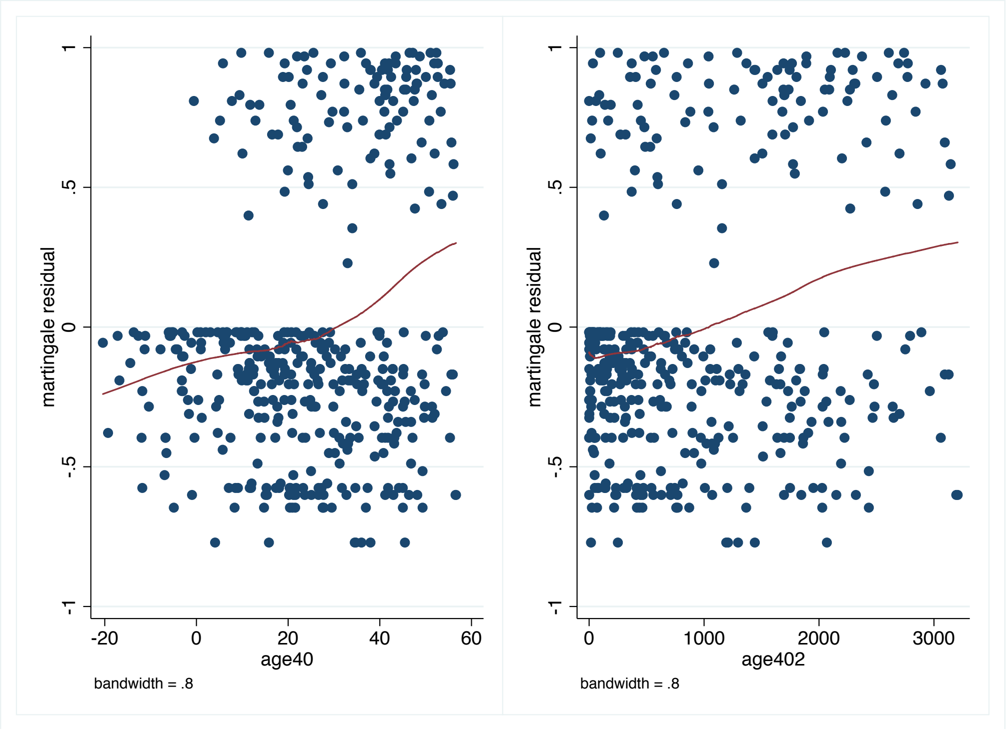

The improved model fit resulting from this change was confirmed by estimating the empirical Nelson-Aalen cumulative hazard function using the Cox-Snell residuals as the time variable. The predictive power of the Cox model was calculated using Harrell's C concordance statistic at 0.75. We conducted leverage analysis using DFBETA, likelihood displacement values, and LMAX, which identified two influential patients. Their exclusion from the analysis did not change our findings.

Finally, we stratified our model by categories of age (<60 and ≥ 60 years; Model A), sex (Model B), frailty (Clinical Frailty Score <4 and ≥4; Model C) and ethnicity (White, Black, Asian, Mixed or other; Model D) to allow for different baseline hazards and compared it to the unstratified Model O. The resulting variable coefficients are shown in Table S2 and we concluded that stratification did not lead to major changes in their value. As a result, we retained simpler Model O as the final model in our Cox survival analysis.

Table S2: Summary of variable coefficient values for four different model stratification choices (Models A-D) compared with the unstratified model (Model O)

	**Model A**	**Model B**	**Model C**	**Model D**	**Model O**
Age (per decade)	-	1.068***	1.067***	1.058***	1.068***
Sex	2.274***	-	2.303***	2.254***	2.306***
CRP (per 10mg/L)	1.028***	1.029***	1.028**	1.029***	1.029***
Presence of CKD	1.881**	1.927**	1.816*	1.914**	1.870**
Presence of Dyspnoea	1.887**	1.897**	1.929**	1.980**	1.881**

* p<0.05; ** p<0.01; *** p<0.001

We subsequently included the same covariates in a parametric survival model. We tested the fit of exponential, Weibul, Gompertz, Lognormal, Loglogistic and generalized gamma distributions and the results of the comparison are shown in Table S3.

Table S3: Comparison of six different parametric models to the Cox model

Model	N	Log Likelihood (null)	Log Likelihood (model)	Degrees of freedom	AIC	BIC
Exponential	427	-333.7153	-296.4073	6	604.8146	629.1553
Weibul	427	-333.3668	-296.3631	7	606.7261	635.1236
Gompertz	427	-330.4032	-295.5255	7	605.0509	633.4484
Lognormal	427	-326.4666	-292.3062	7	598.6124	627.0099
Loglogistic	427	-330.1294	-294.125	7	602.25	630.6475
Gen. gamma	427	-325.0262	-292.2981	8	600.5962	633.0505
Cox	427	-585.6825	-551.624	5	1113.248	1133.532

Based on the AIC, the optimal distribution choice was determined to be the Lognormal. In the accelerated failure-time (AFT) parameterization, the resulting regression equation is the following:

$$\ln(t_j) = \beta_0 + \beta_1 age_j + \beta_2 sex_j + \beta_3 CRP_j + \beta_4 CKD_j + \beta_5 dyspnea_j + u_j$$

where u_j follows a standard normal distribution with mean 0 and standard deviation σ.
Since the lognormal model can only be parameterized in the AFT metric, the coefficients β_0 to β_5 in the previous equation represent Time Ratios, not Hazard Ratios and their values and 95% CI are shown in Table S4 below.

Table S4: Results from the lognormal parametric survival model

Coefficient	Time Ratio	z	P> |z|	95% CI	
β_0	291.958	17.29	0.000	153.384	555.724
β_1	0.935	-5.43	0.000	0.913	0.958
β_2	0.463	-3.60	0.000	0.305	0.704
β_3	0.968	-3.31	0.001	0.950	0.987
β_4	0.561	-2.27	0.023	0.341	0.923
β_5	0.524	-3.04	0.002	0.346	0.795

As described previously, we plotted Martingale and Cox-Snell residuals to visually assess model fit and the results are shown in Figure S7.

Figure S7: Assessment of model fit using Martingale (left) and Cox-Snell (right) residuals

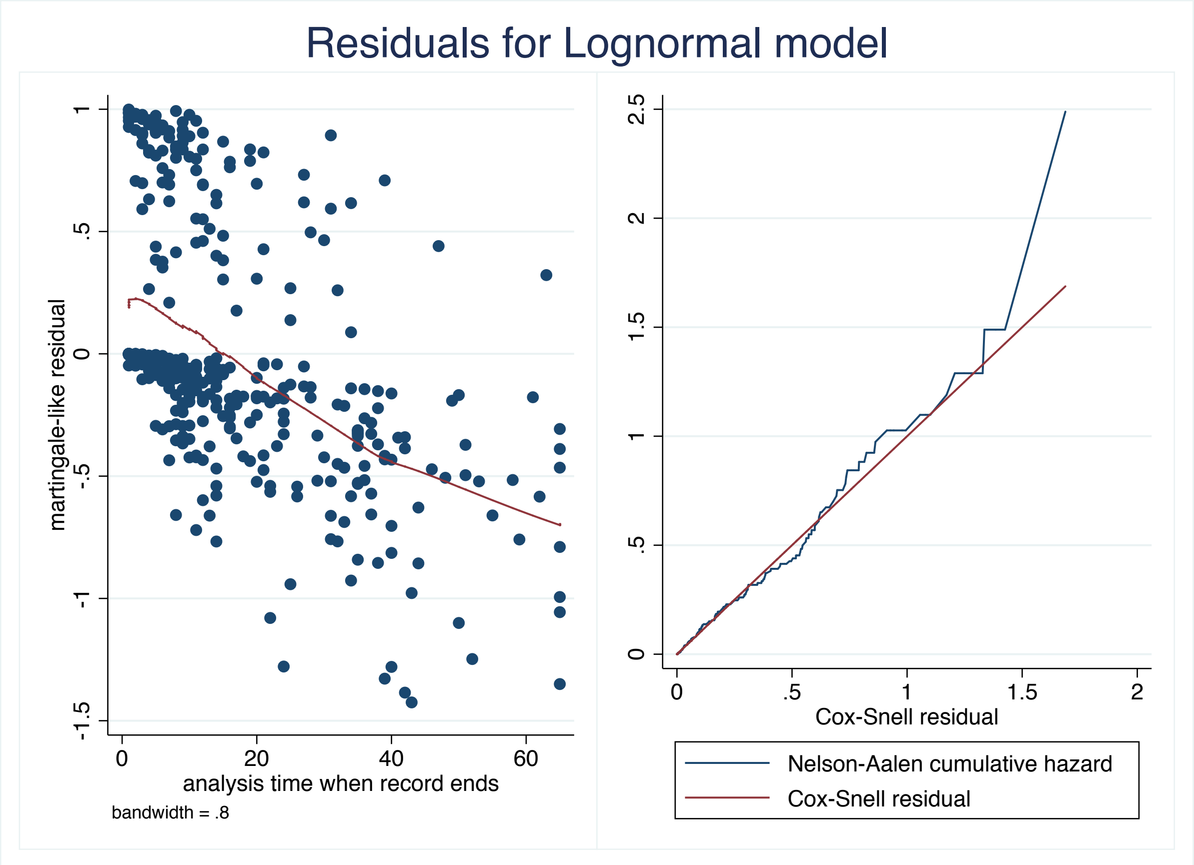

B. Survival analysis

Figure S8: The effect of individual factors on the hazard of death at hospital discharge or 30 days, based on parametric survival analysis. The baseline hazard represents the mortality hazard of a 40-year old female patient with normal C-reactive protein levels (CRP <5 mg/L), no Chronic Kidney Disease (CKD) and no dyspnea on presentation. The additional effect of each factor is modelled individually, with all other covariates held at their baseline value.

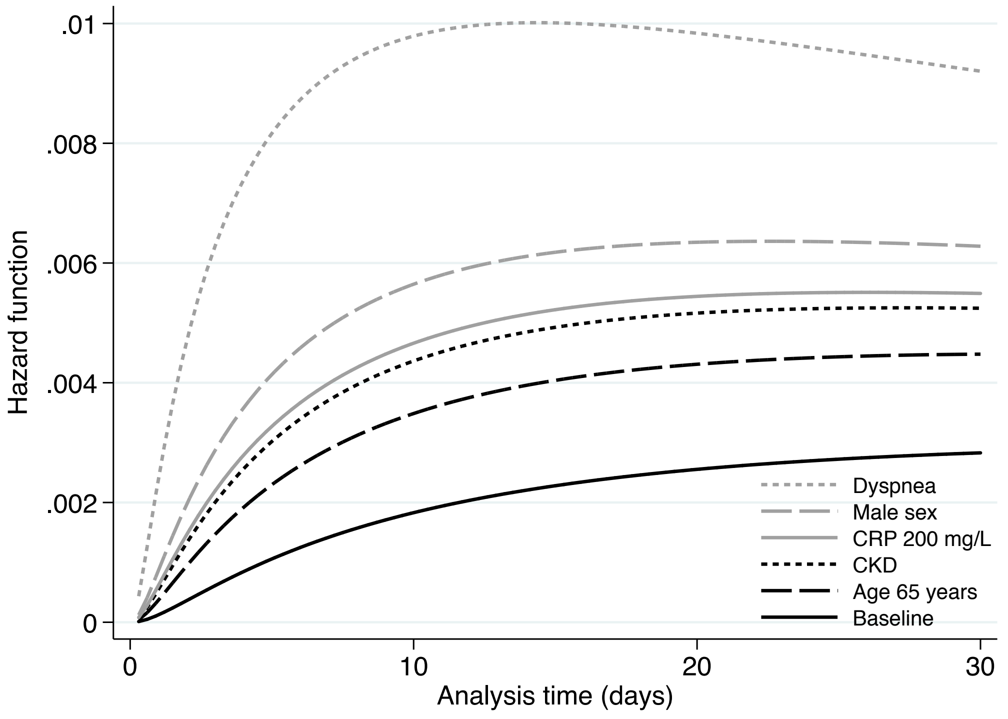

Page intentionally left blank

Hospital mortality and resource implications of hospitalisation with COVID-19 in London, UK: a prospective cohort study

Supplementary File 4

Institutional surge planning and resource implications

KCH implemented a coordinated institutional pandemic surge plan, in collaboration with all hospital departments and the local care network. The main points of this plan and the implications on resource utilization were the following:

i. *Capacity expansion:* during the study period the 950-bed main site at Denmark Hill expanded its effective adult Intensive Care Unit (ICU) capacity from 69 to 129 beds. This included expansion of areas where adult ICU services could be provided from the initial 4 adult ICUs to 3 new areas (a High-Dependency Unit, a post-operative Recovery area and the Paediatric ICU). Initially, one of the ICUs functioned as a dedicated COVID-19 ICU. As the number of cases increased, we implemented reverse-isolation of non-COVID-19 patients and opened areas where respiratory interventions like CPAP could be safely provided under the supervision of Respiratory Medicine physicians. Achieving a similar expansion of capacity over a few weeks required extraordinary effort, coordination and resources.

ii. *Human resources and staffing:* The increased demand for healthcare services coincided with increased health risks to healthcare workers, in circumstances of pre-existing staff shortages. In response, KCH implemented a programme of training, role expansion and redeployment of staff from Anaesthetics, Theatres and other departments to ICU. This included
    a. medical personnel of all grades
    b. nursing staff
    c. allied health professions
    d. supporting and administrative staff.

    Permanent and redeployed staff underwent extensive training on safe practice with simulation sessions. Redeployed staff were embedded into a number of teams: Prone positioning, Intubation, Transfer, and Family communication. These groups underwent more focused training and increased overall efficiency. All staff were supported with the development of new guidelines and protocols. Hospital reorganization of this scale required significant work and was only sustainable in the context of cessation of all programmed activity.

iii. *Equipment and Medicines:* in order to achieve a significant increase in service provision capability on short notice, ICU loaned equipment and expertise from other departments. This included mechanical ventilators, infusion pumps, renal replacement therapy machines, expendables and medicines. Importantly, this also required recruitment of technical support and experienced operators from the respective departments.

iv. *Service provision and organization:* In addition to the time and effort devoted to direct provision of critical care services the following were required:
    a. reconfiguration of the entire service delivery model, including staffing and operating of 3 new ICUs
    b. development of protocols, guidelines, and training material
    c. training of new and existing staff
    d. quality assurance and governance under unprecedented conditions

    This required expenditure of a significant number of official and unofficial working hours, over a prolonged period of time.

    Implementation of the pandemic surge plan resulted in an organized approach towards an unprecedented set of circumstances with substantial resource implications.
